# Unexpected rupture of the left ventricular wall following surgical repair of post-infarction ventricular septal rupture in a patient supported by Impella CP: a case report

**DOI:** 10.1093/ehjcr/ytag054

**Published:** 2026-01-25

**Authors:** Risa Nishio, Yusuke Takei, Yuya Nozawa, Toshiyuki Kuwata, Ikuko Shibasaki

**Affiliations:** Division of Cardiovascular Medicine, Japanese Red Cross Maebashi Hospital, 389-1 Asakura-machi, Maebashi, Gunma 371-0811, Japan; Division of Cardiovascular Surgery, Japanese Red Cross Maebashi Hospital, 389-1 Asakura-machi, Maebashi, Gunma 371-0811, Japan; Department of Cardiac and Vascular Surgery, Dokkyo Medical University School of Medicine, 880 Kitakobayashi, Mibu-machi, Shimotuga-gun, Tochigi 321-0293, Japan; Division of Cardiovascular Surgery, Japanese Red Cross Maebashi Hospital, 389-1 Asakura-machi, Maebashi, Gunma 371-0811, Japan; Division of Cardiovascular Surgery, Japanese Red Cross Maebashi Hospital, 389-1 Asakura-machi, Maebashi, Gunma 371-0811, Japan; Department of Cardiac and Vascular Surgery, Dokkyo Medical University School of Medicine, 880 Kitakobayashi, Mibu-machi, Shimotuga-gun, Tochigi 321-0293, Japan

**Keywords:** Case report, Percutaneous ventricular assist devices, Impella CP, Ventricular septal rupture, Mechanical circulatory support, Left ventricular perforation

## Abstract

**Background:**

Percutaneous ventricular assist devices (pVADs) are important systems in the management of cardiogenic shock, providing effective left ventricular (LV) unloading and enabling preoperative stabilization in critically ill patients. Specifically, their use has expanded to postinfarction ventricular septal rupture (VSR), allowing for delayed surgical intervention once myocardial fibrosis progresses. Nonetheless, mechanical complications associated with prolonged pVAD support, including LV perforation, are rare but potentially fatal.

**Case summary:**

A 70-year-old woman developed VSR following an anterior myocardial infarction involving the left anterior descending artery. Preoperatively, the patient was supported with an Impella CP for 7 days. Although the device functioned normally, frequent premature ventricular contractions were noted during support. The patient underwent surgical VSR repair via the right ventricular approach using the extended sandwich patch technique. The Impella CP was removed before aortic cross-clamping. After patch closure and successful weaning from the cardiopulmonary bypass, sudden bleeding was observed in the posterior LV free wall. Re-arrest occurred, and surgical inspection revealed a 1.5-cm subepicardial rupture in the posterior wall, suspected to have resulted from mechanical contact with the Impella catheter tip. The rupture site was repaired using felt-reinforced sutures and surgical sealant. The patient later required temporary mechanical support due to low-output syndrome, but recovered gradually with preserved LV function and no VSR recurrence.

**Discussion:**

Even with a pigtail-tipped pVAD, inadequate positioning and prolonged support can lead to LV wall injuries. Careful monitoring, positional assessment, and surgical awareness are essential to avoid such complications.

Learning points

- Left ventricular perforation is a rare but life-threatening complication of Impella CP, even in devices with pigtail tips.
- Daily imaging surveillance using echocardiography or fluoroscopy, along with close monitoring of arrhythmias, is essential, particularly during prolonged preoperative support.
- Awareness of cumulative mechanical stress during surgical repair of delayed post-infarction ventricular septal rupture is critical to prevent catastrophic ventricular wall rupture.

## Introduction

Percutaneous ventricular assist devices (pVADs) are mechanical circulatory support (MCS) systems that have been widely adopted recently for cardiogenic shock management.^1^ Notably, their ability to provide stable circulatory support while effectively unloading the left ventricle has attracted attention to pVADs as a new option alongside conventional modalities, such as the intra-aortic balloon pump (IABP) and extracorporeal membrane oxygenation (ECMO). This feature has influenced strategies for addressing post-infarction ventricular septal rupture (VSR), a catastrophic acute myocardial infarction complication.^2,3^ Acute-phase surgical repair is often challenging because of myocardial fragility, which contributes to high mortality and recurrence rates. pVAD support stabilizes haemodynamics and allows for infarct maturation and fibrosis, enabling delayed surgical repair. Recent reports suggest favourable outcomes with this strategy.^4^ However, pVAD-related complications include bleeding, thromboembolic events, and infections.^5^ Left ventricular (LV) free wall perforation immediately after surgical repair is extremely rare,^6^ but should be considered in future treatment strategies and perioperative management. We present a case of intraoperative LV free wall rupture after surgical VSR repair in a patient who was supported preoperatively with Impella CP (Abiomed, Danvers, MA, USA).

## Summary figure

**Figure:**
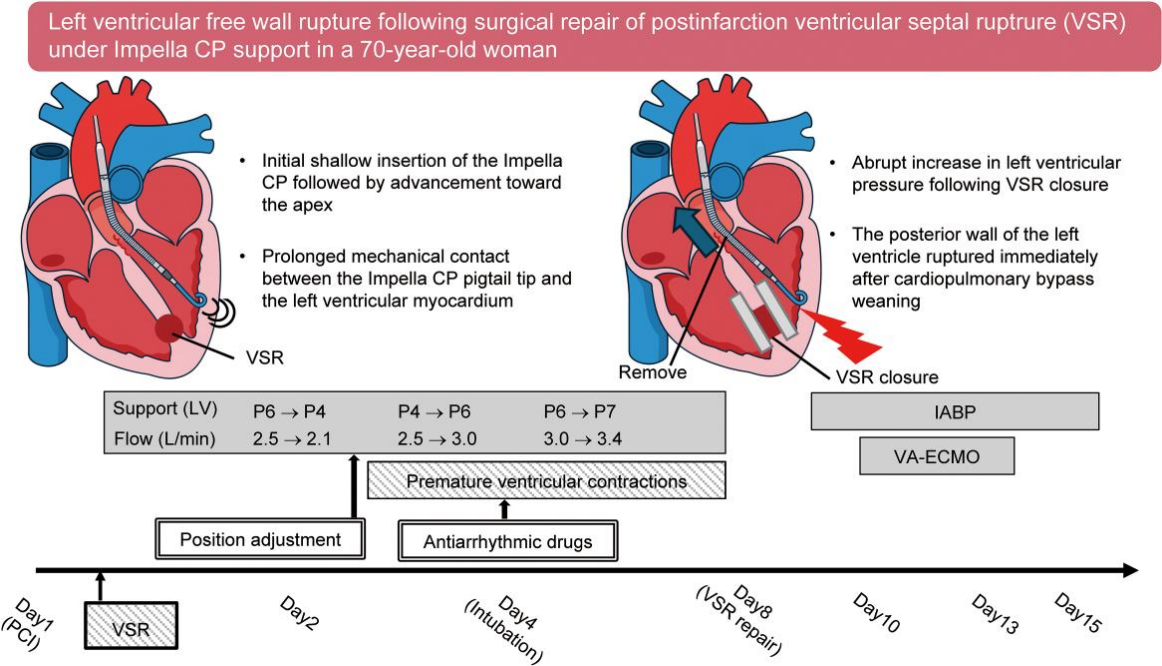


## Case presentation

A 70-year-old woman with no significant medical history developed chest tightness that progressed to persistent chest pain and vomiting. At a local hospital, electrocardiography revealed ST-segment elevation at V2–V5, and she was transferred to our hospital for evaluation and management.

**Figure 1:**
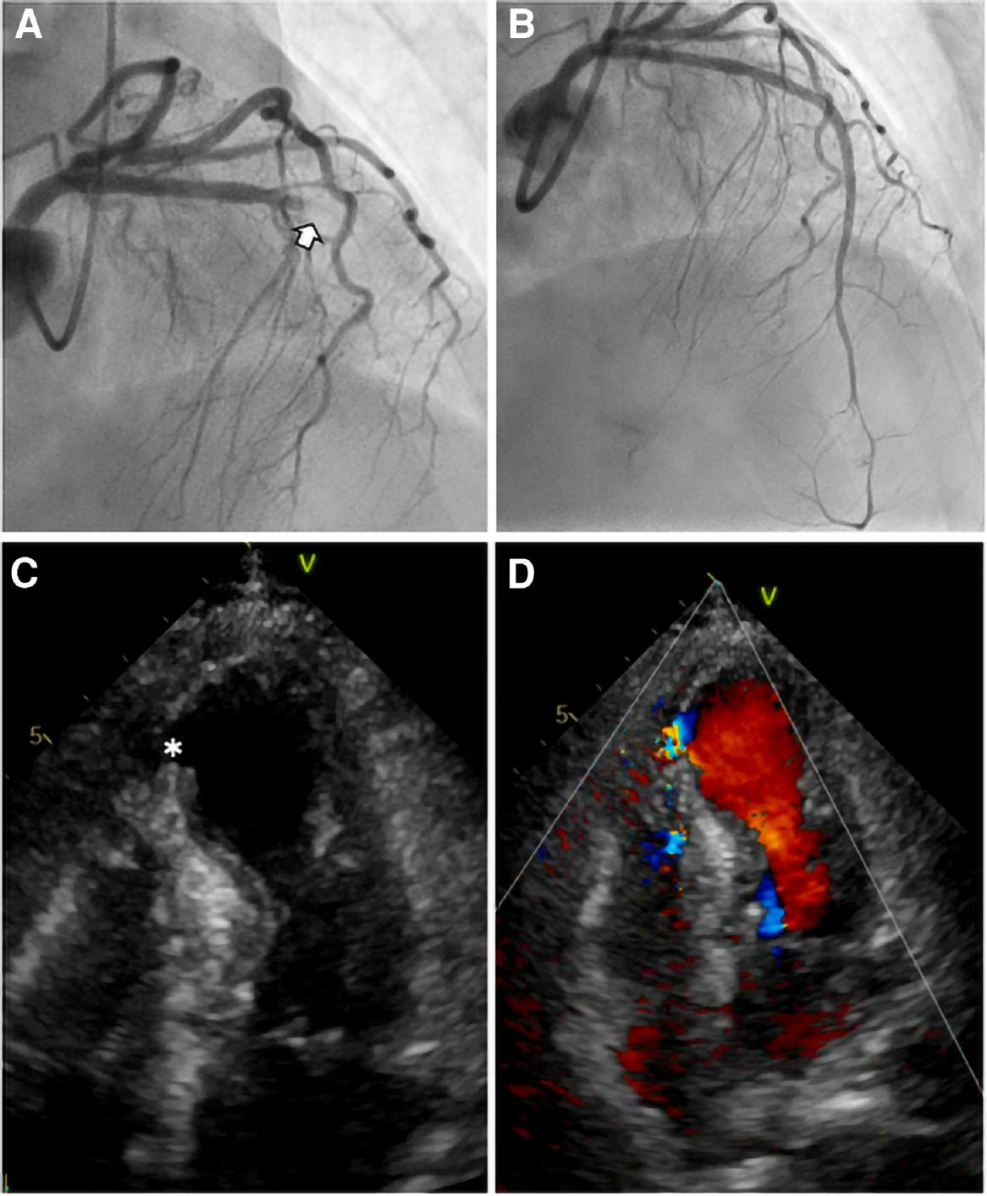
Imaging of acute left anterior descending artery infarction and ventricular septal rupture. (*A*) Coronary angiography showing total left anterior descending artery occlusion (arrow). (*B*) Successful revascularization following drug-eluting stent implantation. (*C*) Transthoracic echocardiography revealing an apical ventricular septal rupture (asterisk). (*D*) Colour Doppler image showing a left-to-right shunt through the ventricular septal rupture.

On arrival, she was haemodynamically stable. Transthoracic echocardiography (TTE) demonstrated preserved LV ejection fraction (approximately 50%) with apical and mid-anterior septal wall akinesis. Labs revealed elevated troponin I (1.78 ng/mL), creatine kinase (688 U/L), and creatine kinase-MB (135.9 U/L) levels. Emergency coronary angiography demonstrated total proximal left anterior descending artery occlusion, which was treated by drug-eluting stent implantation (*Figure 1A, B*).

**Figure 2:**
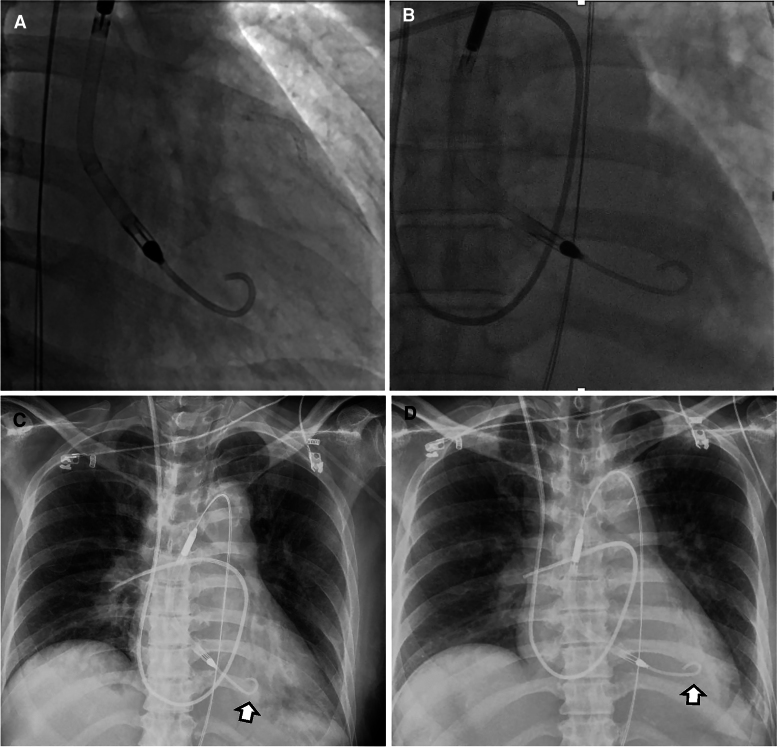
Fluoroscopic and radiographic imaging of Impella CP positioning. (*A*, *B*) Fluoroscopic images during initial placement (*A*) and after repositioning (*B*). (*C*, *D*) Chest radiographs showing Impella CP tip (arrows) before (*C*) and after (*D*) repositioning. The device was initially placed in a shallow position due to arrhythmias suspected to result from myocardial wall contact. Repositioning advanced the catheter slightly, orienting the tip towards the posterior wall.

Subsequently, hypotension and an elevated lactate level (4.0 mmol/L) developed. A new systolic murmur and repeat TTE revealed a VSR near the apical septum (*Figure 1C, D*). Following a multidisciplinary heart team discussion, a delayed surgical repair strategy was selected to allow for haemodynamic stabilization and infarct remodelling. Impella CP was used as the MCS. The device was initially placed in a shallow position owing to premature ventricular contractions, likely caused by myocardial wall contact. However, unstable flow support and frequent suction alarms prompted repositioning, resulting in advancement of the tip towards the posterior wall (*Figure 2*, *Video 1*). Stable support was achieved at P6–P7 (3.0–3.4 L/min). By day 4, premature ventricular contractions and non-sustained ventricular arrhythmias were observed. Antiarrhythmic therapy began with intravenous lidocaine and landiolol. Sedation and intubation followed due to increasing restlessness. The Impella CP tip was poorly visualized on TTE, and appeared directed towards the posterior wall (*Video 2*). As flow support remained stable and the arrhythmias were controlled, no further repositioning was performed. Surgical repair was performed on day 8.

**Figure 3:**
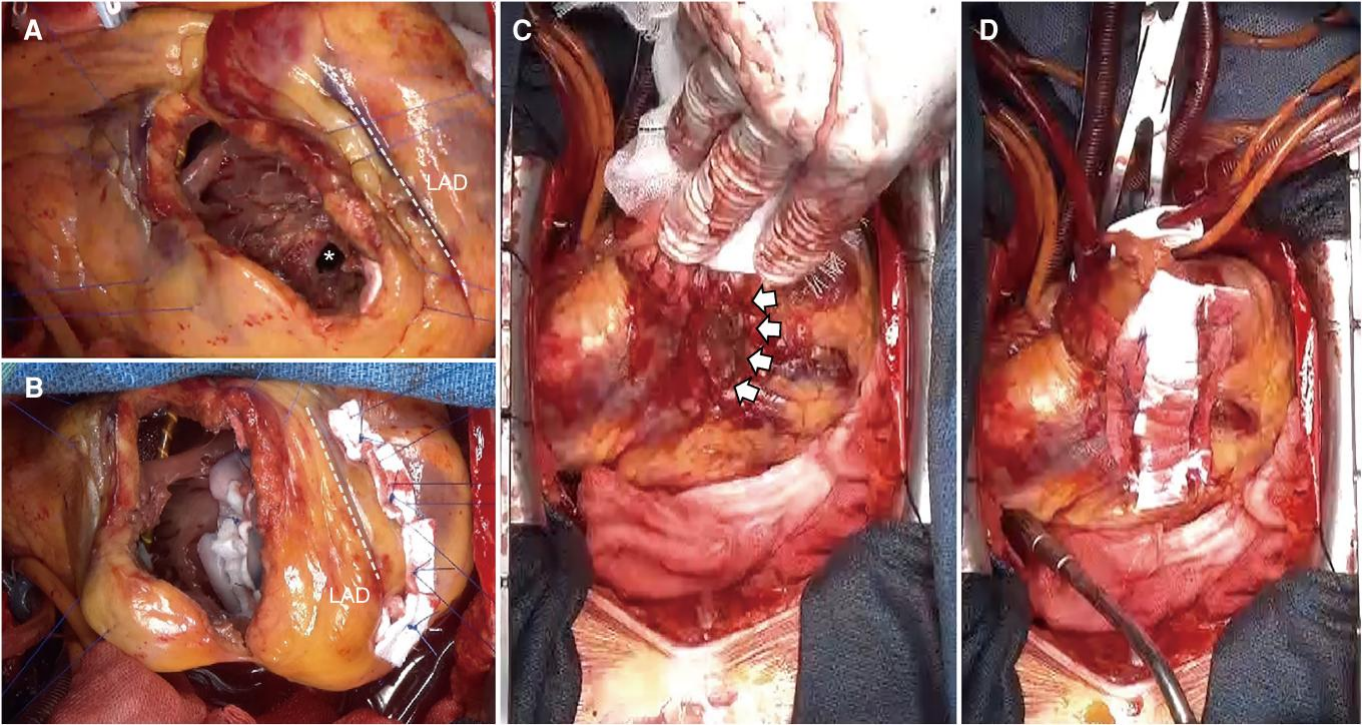
Surgical findings and management of left ventricular wall rupture. (*A*) Right ventriculotomy was performed, and the ventricular septal rupture was identified (asterisk) near the apex, measuring approximately 2 cm × 1 cm. (*B*) Ventricular septal rupture repair was performed using two octagonal bovine pericardial patches (6 cm in diameter). (*C*) Following attempted weaning from cardiopulmonary bypass, sudden bleeding from the posterior aspect of the heart was observed. Elevation of the heart revealed a 1.5-cm rupture site (arrows) in the posterior wall. (*D*) The rupture site was repaired by debridement of the necrotic myocardium and closure using felt strips and 3-0 polypropylene mattress sutures. Abbreviations: LAD, left anterior descending artery.

The VSR was repaired using an extended sandwich patch technique.^7^ Following cardiopulmonary bypass (CPB), the Impella CP was removed before aortic cross-clamping. Cardioplegia was administered antegrade and retrograde. A 5-cm right ventriculotomy was performed 1.5 cm lateral and parallel to the left anterior descending artery. The VSR, measuring approximately 2 × 1 cm, was identified in the anterior portion of the ventricular septum near the apex (*Figure 3A*). VSR closure was achieved using two octagonal bovine pericardial patches (*Figure 3B*). The right ventriculotomy was closed with felt-reinforced sutures. Transoesophageal echocardiography confirmed successful VSR closure. However, sudden pericardial bleeding was observed during preparation for decannulation. Elevation of the cardiac apex revealed active bleeding from the apical and posterior walls. CPB was immediately reinstituted, and cardiac arrest was reestablished. Inspection identified a 1.5-cm rupture in the subepicardial region of the posterior wall (*Figure 3C*). Initial haemostasis by direct closure with simple sutures and Teflon-felt reinforcement failed due to extreme myocardial friability. Necrotic tissue was carefully debrided, and the fragile LV wall was reinforced and closed with felt strip sutures (*Figure 3D*, *Video 3*). The patient was successfully weaned from CPB. With stable haemodynamics, an IABP was placed for LV unloading, instead of reinserting a new pVAD.

On postoperative day 2, the patient developed low-output syndrome and required veno-arterial ECMO. As the Impella CP was suspected of being associated with the prior LV rupture, the catheter was not reinserted. LV function gradually recovered, allowing ECMO and IABP removal on postoperative days 5 and 7, respectively. The patient was discharged after rehabilitation. At the 6-month follow-up, TTE demonstrated preserved cardiac function, with an LV ejection fraction of 60% and no VSR recurrence.

## Discussion

The 2023 ESC Guidelines^8^ recognize the emerging role of temporary MCS in pre-operative or prophylactic support for the mechanical complications of acute coronary syndrome, although high-level evidence remains limited. In this case, Impella CP was employed to stabilize haemodynamics and allow for delayed surgical repair.

LV perforation or rupture with pVADs is rare, but potentially fatal, with an incidence of approximately 0.2%.^5^ Most reported cases occur during device insertion and are attributed to direct myocardial trauma from the device tip or guidewire.^9,10^ LV perforation during VSR repair has been reported with Impella 5.5, attributed to its rigid shaft and lack of a pigtail tip, which can cause direct myocardial penetration.^6^ A similar injury has been described in fulminant myocarditis, particularly with deep placement of the Impella 5.5.^11^

In contrast, our case involved the pigtail-tipped Impella CP. Although rupture occurred near the presumed catheter tip, the absence of bleeding upon device removal suggested that prolonged mechanical contact during support had compromised the posterior wall, which subsequently may have failed under sudden pressure overload following VSR closure. Post-repositioning fluoroscopy and chest radiography showed the pigtail curving towards the posterior wall, and limited TTE visualization suggested a similar orientation. Patient movement before intubation may have altered the Impella CP position. Frequent ventricular arrhythmias—potential early signs of myocardial irritation—prompted intubation and sedation. Antiarrhythmic therapy temporarily resolved the arrhythmias, possibly masking their warning significance.

This case underscores that even Impella CP, despite its softer pigtail tip, can cause mechanical injury when positioned deeply or inadequately.

It also provides clinical insights into the management of VSR patients with pVADs. First, routine monitoring of device position is essential, as spontaneous shifts may occur before delayed VSR repair. Daily chest radiography and TTE can detect malposition early, and recurrent arrhythmias during support may indicate abnormal myocardial contact—even when device performance appears normal—and should prompt imaging.

Second, the overall strategy for pVAD use should be individualized to the patient’s condition and surgical plan. In this case, Impella CP was selected preoperatively due to limited organ dysfunction and removed before cross-clamping in line with its recommended usage duration. Since the rupture was attributed to the device, we avoided reinserting a pVAD, and IABP was chosen postoperatively. When pVADs are used intraoperatively or reinserted, careful adjustment of depth and angulation is crucial. Surgical approach also influences mechanical injury risk; the right ventricular approach avoids incising fragile myocardium but limits direct visualization of the device, increasing the chance of unrecognized displacement during cardiac manipulation.

In conclusion, as pVADs are increasingly employed for postinfarction VSR, vigilant position monitoring, individualized support strategies, and intraoperative awareness are critical to prevent mechanical complications.

## Lead author biography


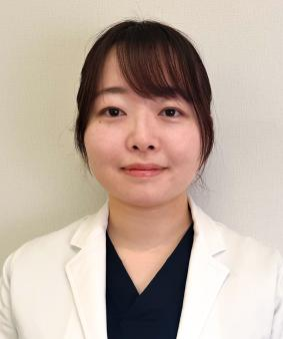


Dr. Risa Nishio is a resident in cardiology at the Japanese Red Cross Maebashi Hospital, Japan. She received her M.D. from Akita University in 2020 and completed her initial clinical training at the Japanese Red Cross Maebashi Hospital. She is currently undergoing an advanced residency in cardiology at the same institution, with clinical interests in mechanical circulatory support, cardiovascular intervention, and care.

## Authors contributions

Risa Nishio (Investigation, Writing—original draft, Visualization, Resources), Yusuke Takei (Conceptualization, Project administration, Writing—original draft, Writing—review & editing, Visualization), Yuya Nozawa (Resources), Toshiyuki Kuwata (Resources), Ikuko Shibasaki (Writing—review & editing, Supervision)

## Data Availability

The datasets generated and/or analysed in the current study are available from the corresponding author upon reasonable request.

## Notes

**Consent:** The authors confirm that written consent for submission and publication of this case report including images and associated text has been obtained from the patient, in line with COPE guidance.
